# Associations of Early Life Ambient PM_2.5_ Exposure With Asthma Risk in a Cohort of Preterm Infants With Bronchopulmonary Dysplasia

**DOI:** 10.1002/ppul.71432

**Published:** 2025-12-17

**Authors:** Lizbeth F. Gomez, Jonathan J. Szeto, Joshua K. Radack, Nicolas P. Novick‐Goldstein, Kristan A. Scott, Daria C. Murosko, Kathleen A. Gibbs, Ella Whitman, Jonathan C. Levin, Scott A. Lorch, Sara B. DeMauro, Chén C. Kenyon, Allan C. Just, Heather H. Burris, Timothy D. Nelin

**Affiliations:** ^1^ Division of Pulmonary, Allergy, and Critical Care of Medicine University of Pennsylvania Philadelphia Pennsylvania USA; ^2^ The Palliative and Advanced Illness Research (PAIR) Center Perelman School of Medicine Philadelphia Pennsylvania USA; ^3^ Department of Pediatrics Children's Hospital of Philadelphia and University of Pennsylvania Philadelphia Pennsylvania USA; ^4^ Leonard Davis Institute of Health Economics Philadelphia Pennsylvania USA; ^5^ Robert Larner College of Medicine University of Vermont Burington Vermont USA; ^6^ Department of Pediatrics, Divisions of Newborn and Pulmonary Medicine Harvard Medicine School Boston Massachusetts USA; ^7^ Department of Epidemiology Brown University Providence Rhode Island USA; ^8^ Center of Excellence in Environmental Toxicology, Perelman School of Medicine University of Pennsylvania Philadelphia Pennsylvania USA

## Abstract

**Objective:**

To examine whether exposure to fine particulate matter (PM_2.5_) during the first year after neonatal intensive care unit (NICU) discharge is associated with asthma by age 5 among infants with bronchopulmonary dysplasia (BPD).

**Methods:**

We conducted a retrospective cohort study of 337 infants with BPD, born between 2010 and 2019, who survived to discharge with clinical follow‐up in the Children's Hospital of Philadelphia Care Network through age 5. Daily residential census block group PM_2.5_ exposures were estimated using a spatiotemporal machine‐learning model and averaged over the first year after NICU discharge. Modified Poisson regression models with robust standard errors quantified associations of PM2.5 with asthma by age 5, adjusting for neonatal clinical factors, insurance, neighborhood deprivation, and race/ethnicity.

**Results:**

By age 5 years, 169 (50.1%) infants had an asthma diagnosis. Mean annual PM_2.5_ exposure was 8.8 µg/m^3^ (SD 1.1). Each 1 µg/m^3^ increment of PM_2.5_ was associated with higher asthma risk (unadjusted RR 1.14, 95% CI: 1.03–1.25; fully adjusted aRR 1.19, 95% CI: 1.03–1.37). Compared to the lowest exposure tertile (mean 7.6 µg/m^3^), adjusted rates of asthma tended to be higher as exposure increased: Tertile 2 (mean 8.7 µg/m^3^, aRR 1.31; 95% CI: 0.98–1.74), Tertile 3 (mean 10.0 µg/m^3^, aRR 1.68, 95% CI: 1.17–2.4).

**Conclusions:**

Exposure to higher ambient PM_2.5_ in the year after NICU discharge was associated with asthma by age 5 among children with BPD. These findings highlight early‐life air quality as a modifiable determinant of long‐term respiratory outcomes in infants with BPD.

## 1 Introduction

Bronchopulmonary dysplasia (BPD) is a chronic lung disease primarily affecting preterm infants who require prolonged ventilatory support in the neonatal intensive care unit (NICU) [1, 2]. Among infants born extremely preterm (< 28 weeks of gestation), BPD represents the most prevalent chronic complication, affecting approximately half of survivors [3]. BPD is characterized by disrupted alveolar development and increased airway reactivity [4], leading to persistent pulmonary deficits that can extend into childhood [5]. As children with BPD grow, they are at significantly elevated risk of chronic respiratory morbidities, including recurrent wheezing, respiratory infections, and asthma [5, 6]. Notably, nearly half of infants with BPD require hospital readmission for respiratory illness within their first year after discharge, which highlights the significant respiratory vulnerability these infants face in the early post‐discharge period [1, 7, 8, 9]. Furthermore, as BPD impairs alveolarization and heightens airway reactivity, ambient air pollution represents a potential stressor and contributor to respiratory illness.

Exposure to ambient air pollutants, such as particulate matter with an aerodynamic diameter of ≤ 2.5 µm (PM_2.5_), has been extensively associated with adverse respiratory outcomes in children [9, 10, 11, 12, 13, 14]. Ambient PM_2.5_ can penetrate the pulmonary system, and thus, elevated levels have been linked to higher rates of asthma exacerbations and hospitalizations, inflammatory responses, impaired lung function, and disruption of normal respiratory development [15, 16]. While the general pediatric population is susceptible to ambient air pollution, infants with BPD may represent an especially vulnerable subgroup, given prolonged supplemental respiratory support requirement in the setting of pulmonary underdevelopment, inflammation, and damage. Consequently, even modest increments in PM_2.5_ exposure could exacerbate preexisting lung deficits and increase risk for subsequent respiratory complications [9, 14, 17, 18].

Understanding the role of environmental exposures during critical developmental windows is essential for mitigating long‐term respiratory risks in vulnerable populations. For infants and children with BPD, the year following NICU discharge represents a pivotal phase in lung maturation, characterized by rapid alveolar and vascular growth, remodeling, and recovery from neonatal injuries [19]. Although biologically plausible, evidence directly linking early‐life PM_2.5_ exposure to asthma risk among children with BPD remains limited, and this knowledge gap may hinder the development of targeted preventive strategies aimed at improving long‐term respiratory outcomes in this vulnerable population.

In this study, we evaluated whether higher mean ambient PM_2.5_ exposure in the first 12 months following hospital discharge is associated with asthma diagnosis at age 5 among children previously diagnosed with BPD. We hypothesized that elevated PM_2.5_ exposure during this critical postnatal window would be associated with a higher risk of asthma onset by age 5.

## 2 Methods

### 2.1 Study Design and Population

We conducted a retrospective cohort study using electronic health records (EHR) of infants born at less than 32 weeks' gestation and diagnosed with BPD. Infants included in this analysis received neonatal care at the Children's Hospital of Philadelphia (CHOP) or the University of Pennsylvania Health System between January 1, 2010, and December 31, 2019. The diagnosis of BPD was determined based on criteria defined by the 2019 Neonatal Research Network (NRN) [20] which classifies BPD severity into three distinct grades according to the level of respiratory support received at 36 weeks postmenstrual age: Grade 1 involves nasal cannula at a flow of ≤ 2 L/min; Grade 2 involves nasal cannula at a flow of > 2 L/min or noninvasive positive airway pressure; and Grade 3 involves invasive mechanical ventilation. We included infants who survived until hospital discharge and who had residential information documented in the metropolitan Philadelphia region for the first 12 months following NICU discharge. We used residential data to estimate post‐discharge ambient air pollutant exposure at the census block group level. To ensure adequate clinical follow‐up and ascertain asthma diagnosis, we further restricted to infants with documented clinical follow‐up within the CHOP Care Network occurring through age 5; each included child had a least one outpatient visit after their fifth birthday. CHOP maintains over 30 pediatric primary care practices and over 15 specialty care and surgery centers throughout Pennsylvania and New Jersey [21]. Infants were included if they received any type of care including primary care, subspecialty care, acute care (e.g., urgent care or emergency department), or inpatient care at any CHOP‐affiliated site through age five. This allowed sufficient follow‐up time to reliably capture asthma diagnoses within the EHR. After applying these inclusion criteria, the final analytic cohort consisted of 337 infants. Characteristics comparing the included and excluded infants are displayed in Supporting Information S1: Table 1.

### 2.2 Outcome

The primary outcome was diagnosis of asthma by 5 years of age. Asthma diagnoses were identified through a two‐step process. First, asthma diagnoses were defined as at least one documented diagnosis using the International Classification of Diseases, Tenth Revision (ICD‐9/10), asthma codes (493.x/J45.x, available in Supporting Information S1: Table 2). Second, to enhance diagnostic accuracy, we then conducted manual chart reviews within the Epic electronic health system, using the search bar function to query for the term “asthma” across clinical documentation including provider notes, associated diagnoses for medication prescriptions, and clinical encounters. Chart review was performed by two independent reviewers (J.J.Z. and T.D.N.), with discrepancies resolved by consensus. Asthma diagnosis was assigned to infants who met either ICD‐9/10 or chart review criteria. All infants who were assessed to have asthma by ICD code also had confirmatory evidence on chart review.

### 2.3 Exposure Assessment

Residential addresses at the time of hospital discharge were extracted from the EHR and geocoded using ArcMap software (version 10.8; Esri, Redlands, CA), with ArcGIS StreetMap Premium North America 2021.1 address locator. We assigned each address to the corresponding census block group using 2010 US Census boundaries and estimated daily ambient PM_2.5_ concentrations with a spatiotemporal model that incorporates extreme gradient boosting and inverse‐distance weighting XGBoost‐IDW Synthesis (XIS) [23]. The XIS model uses satellite aerosol optic depth, meteorological, land use, and topographical parameters, yielding high‐resolution validated estimates across the contiguous United States from 2003 to 2023. Daily PM_2.5_ exposure predictions were averaged across the first 12 months post‐NICU discharge. For infants residing at multiple addresses, annual averages were calculated using address‐specific daily estimates accounting for moves. Residential moves and their respective durations were systematically captured and verified via the EHR and assumed to be the midpoint between address changes.

### 2.4 Covariates

We selected confounders a priori based on established associations in the literature and clinical judgment [7, 8, 24, 25, 26, 27, 28]. The relevant clinical neonatal covariates extracted from the EHR were gestational age, birth weight, infant sex, BPD grade, age at NICU discharge, respiratory support at the time of discharge, and inhaled medication use at the time of NICU discharge. In addition, we included birth period (2010–2014; 2015–2019) to reflect trends in PM_2.5_ exposure in the region and the updated National Ambient Air Quality Standard for PM_2.5_ (mean PM_2.5_ by birth year in supplement) [29, 30], infant insurance type (public vs. commercial), census tract‐level neighborhood material community deprivation using a validated measure that incorporates multiple neighborhood socioeconomic indicators [22], and race/ethnicity.

### 2.5 Statistical Analysis

Baseline cohort characteristics were summarized using standard descriptive statistics. Modified Poisson regression models with robust standard errors quantified the association of mean PM_2.5_ exposure during the first 12 months post‐NICU discharge with risk of asthma diagnosis through age 5 among infants with BPD. Regression models were sequentially built with initial adjustment for neonatal clinical covariates, gestational age, birth weight, infant sex, BPD grade, age at NICU discharge, respiratory support at the time of discharge, and inhaled medication use at the time of NICU discharge. Subsequent additional adjustment for insurance type and neighborhood community material deprivation, and final additional adjustment for race/ethnicity. This additive adjustment strategy was used to evaluate the extent to which the association of post‐discharge PM_2.5_ exposure with asthma risk was influenced by individual‐level clinical and sociodemographic factors, and neighborhood characteristics. We added race/ethnicity to the final model given that it is a social construct that serves as a proxy for multiple exposures, which may include exposure to PM_2.5_ [31, 32]. Model assumptions, including linearity of continuous predictors with the log link and potential multicollinearity among covariates, were evaluated prior to final modeling. To examine potential nonlinear exposure–outcome relationships, we categorized annual PM_2.5_ exposure into tertiles and re‐estimated the Poisson regression models, including indicator variables for Tertile 2 and Tertile 3, with Tertile 1 (lowest exposure) serving as the reference category.

In sensitivity analyses, we tested potential bias arising from differential referral patterns and geographic variability in access to clinical sites; we refitted the primary analysis stratifying on residential addresses within and outside of Philadelphia County. All statistical tests were two‐sided, and significance was set at *p* < 0.05. This study was approved by the Institutional Review Board of the Children's Hospital of Philadelphia (IRB #20‐018358). Given the retrospective nature of the study, the IRB did not require informed consent. This study is reported in accordance with the STROBE (Strengthening the Reporting of Observational Studies in Epidemiology) statement, using the checklist for cohort studies to guide transparent reporting. All analyses were conducted using Stata statistical software (Version 18, StataCorp, College Station, TX). Code is available at https://github.com/tnelin/PM2.5_Asthma.git.

## 3 Results

### 3.1 Population Characteristics at Baseline

**Figure 1:**
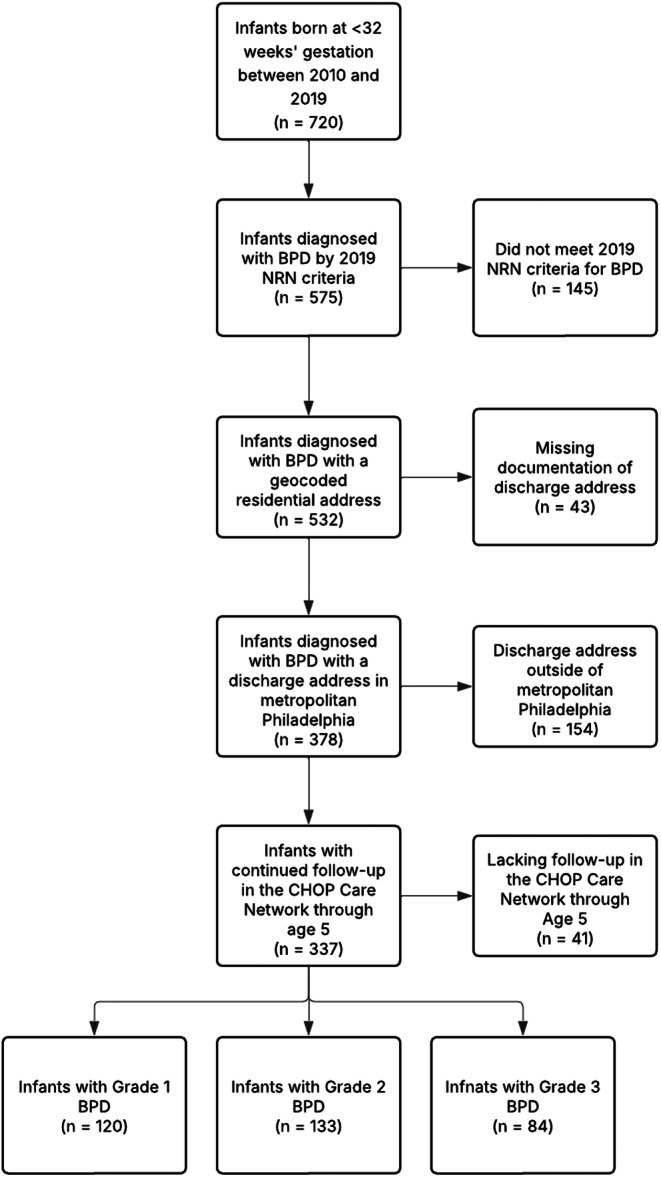
Flowchart of cohort inclusion and exclusion criteria in the participant selection process.

Population characteristics at baseline are shown in Table 1. We identified 337 infants who had a geocoded address and clinical follow‐up in the CHOP Care Network through age 5 (Figure 1). The cohort predominantly included infants who were male (59%), born after 2014 (61%), and with public insurance (62%). Over half were identified as non‐Hispanic Black in the EHR (52%). The median [IQR] gestational age at birth was 27 [25–28] weeks, with the majority classified as extremely preterm (< 28 weeks'). The median [IQR] birth weight was 771 g and approximately three‐quarters of the infants had extremely low birth weights (< 1000 g). At the time of NICU discharge, infants had a mean (SD) age of 156 (89) days. While most infants (72%) were discharged without inhaled respiratory medications, 17% received albuterol alone, and 11% were prescribed albuterol/inhaled corticosteroid in combination. Approximately one‐fourth required some form of respiratory support at discharge, including supplemental oxygen (12%) or tracheostomy (14%). The mean (SD) material community neighborhood deprivation was 0.41 (0.16), and the mean (SD) PM2.5 was 8.8 (1.1) μg/m^3^ (Table 2). Annual mean PM_2.5_ exposure significantly varied by tertile of PM_2.5_ with a mean (SD) PM_2.5_ exposure of 7.6 (0.4) in the lowest tertile and 10.0 (0.6) μg/m^3^ in the highest tertile of exposure, *p *< 0.001 (Table 2). Annual mean PM_2.5_ exposure decreased over the study period (Supporting Information S1: Figure 1). There were 159 infants (47%) with a discharge address in Philadelphia County (Supporting Information S1: Table 3).

**Table 1:** Demographic baseline characteristics of included infants.

Characteristic	Overall	No asthma diagnosis by age 5	Asthma diagnosis by age 5
**Demographic features**	*n* = 337	*n* = 168	*n* = 169
Sex (male)—*n* (%)	199 (59)	99 (59)	100 (59)
Birth period > 2014—*n* (%)	207 (61)	104 (62)	103 (61)
Insurance—*n* (%)			
Commercial	129 (38)	72 (43)	57 (34)
Public	208 (62)	96 (57)	112 (66)
Race/ethnicity—*n* (%)			
Non‐Hispanic Black	174 (52)	75 (44)	99 (59)
Non‐Hispanic White	88 (26)	53 (32)	35 (21)
Other	75 (22)	40 (24)	35 (21)
**Clinical features**			
Gestational age—weeks, median [IQR]	27 [25–28]	27 [25–29]	26 [25–27]
Extremely preterm—*n* (%)	227 (67)	98 (58)	129 (76)
Birth weight—g, median [IQR]	771 [630–1015]	820 [643–1073]	737 [609–930]
ELBW^+^—*n* (%)	249 (74)	114 (68)	135 (80)
BPD Grade—*n* (%)^a^			
Grade 1	120 (36)	64 (38)	56 (33)
Grade 2	133 (39)	62 (37)	71 (42)
Grade 3	84 (25)	42 (25)	42 (25)
Readmission in year after NICU discharge—*n* (%)	164 (49)	57 (34)	107 (63)
Discharge age—days, mean (SD)	156 (89)	154 (93)	158 (86)
Medications at NICU discharge—*n* (%)			
Albuterol	58 (17)	31 (18)	27 (16)
Albuterol/inhaled corticosteroid	37 (11)	11 (6)	26 (15)
Discharge respiratory support—*n* (%)			
No support	251 (74)	127 (76)	124 (73)
Supplemental oxygen	40 (12)	18 (10)	22 (13)
Tracheostomy	46 (14)	23 (14)	23 (13)

*Note:* Extremely preterm infants correspond to infants born before 28 weeks of gestation.

^a^ BPD grade criteria are defined by the 2019 Neonatal Research Network. Grade 1 = nasal cannula at a flow of ≤ 2 L/min; Grade 2 = nasal cannula at a flow of > 2 L/min or noninvasive positive airway pressure; and Grade 3 = to infants requiring invasive mechanical ventilation.

Abbreviations: BPD, bronchopulmonary dysplasia; ELBW, extremely low birth weight < 1000 g; NICU, neonatal intensive care unit.

**Table 2:** Socioenvironmental features of the cohort.

Characteristic	Overall	No asthma diagnosis by age 5	Asthma diagnosis by age 5
	*n* = 337	*n* = 168	*n* = 169
PM_2.5_, mean (SD), µg/m^3^	8.8 (1.1)	8.6 (1.1)	8.9 (1.1)
Neighborhood community material deprivation^a^, mean (SD)	0.41 (0.16)	0.38 (0.16)	0.43 (0.16)

**PM_2.5_ exposure and asthma diagnosis by PM_2.5_ tertile**	**PM**_**2.5**_ **Tertile 1**	**PM**_**2.5**_ **Tertile 2**	**PM**_**2.5**_ **Tertile 3**
	*n* = 113	*n* = 112	*n* = 112
PM_2.5_, mean (SD), µg/m^3^	7.6 (0.4)	8.7 (0.3)	10.0 (0.6)
Neighborhood community material deprivation^a^, mean (SD)	0.38 (0.16)	0.41 (0.15)	0.43 (0.17)
Asthma diagnosis by age 5, *n* (%)	45 (40)	58 (52)	66 (59)

Abbreviation: PM_2.5_, fine particulate matter with aerodynamic diameter ≤ 2.5 μm.

^a^ Six variables from the American Community Survey are used to create the Community Material Deprivation Index: fraction of the population with income in the past 12 months below the poverty level, median household income in the past 12 months, fraction of the population 25 and older with attainment of at least a high school degree, fraction of the population with no health insurance, fraction of households receiving public assistance income or food stamps or SNAP in the past 12 months, and fraction of houses that are vacant [22]. The nationwide mean for the 2018 deprivation index was 0.35. The cohort mean for the 2018 deprivation index in metropolitan Philadelphia was 0.41.

**Table 3:** Modified Poisson regression models quantifying the association of mean annual PM_2.5_ exposure during the first 12 months post‐NICU discharge with asthma diagnosis by age 5 among infants with BPD (*n* = 337).

PM_2.5_ exposure	Model 0	Model 1ᵃ	Model 2ᵇ	Model 3ᶜ
	RR (95% CI)	aRR (95% CI)	aRR (95% CI)	aRR (95% CI)
Continuous, per 1 µg/m^3^ PM_2.5_	1.14 (1.03, 1.25)	1.24 (1.08, 1.42)	1.19 (1.03, 1.37)	1.19 (1.03, 1.37)
Tertile 1	Reference	Reference	Reference	Reference
Tertile 2	1.30 (0.97, 1.74)	1.35 (1.01, 1.81)	1.31 (0.98, 1.74)	1.31 (0.98, 1.74)
Tertile 3	1.48 (1.12, 1.95)	1.81 (1.26, 2.58)	1.68 (1.17, 2.42)	1.68 (1.17, 2.41)

*Note:* Estimates obtained using Poisson regression with robust standard errors.

Abbreviations: aRR, adjusted risk ratio; PM_2.5_, fine particulate matter with aerodynamic diameter ≤ 2.5 μm; RR, risk ratio.

ᵃ Adjusted for gestational age, birth weight, birth period, sex, BPD grade, discharge age, discharge support, and discharge medication.

ᵇ Model 1 + insurance type and census‐tract neighborhood community material deprivation.

ᶜ Model 2 + race/ethnicity.

### 3.2 Asthma by Age 5

By age 5, approximately 50% of the cohort had received a physician diagnosis of asthma. PM_2.5_ exposure in the first 12 months post‐NICU discharge was significantly associated with asthma diagnosis. Each µg/m^3^ increment of PM_2.5_ was associated with 14% higher risk of asthma (RR 1.14; 95% CI: 1.03, 1.25) (Table 3). The association persisted after adjustment for clinical covariates (aRR 1.24; 95% CI: 1.08, 1.42). The risk of asthma remained elevated after adjustment for insurance status, neighborhood community material deprivation, and race/ethnicity (aRR 1.19; 95% CI: 1.03, 1.37) (Table 3).

When PM_2.5_ exposure was modeled in tertiles, we observed a potential exposure dose–response pattern. Compared with infants in the lowest tertile (mean 7.6 µg/m^3^), those in the second tertile (mean 8.7 µg/m^3^) had a modestly elevated radjusted risk of asthma that was significant with adjustment for clinical covariates (aRR 1.35; 95% CI: 1.01, 1.81) but confidence intervals included the null with further adjustment for insurance, neighborhood deprivation, and race/ethnicity (aRR 1.31; 95% CI: 0.98, 1.74). In contrast, compared to the lowest tertile, effect estimates for infants in the highest tertile (mean 10.0 µg/m^3^) were significant in unadjusted and all adjusted models, with 68% higher risk in the fully adjusted model (aRR 1.68; 95% CI: 1.17, 2.41) (Table 3).

Results were similar when the cohort was restricted to within and outside of Philadelphia County; point estimates for continuous exposure were similar for continuous exposures and for the highest tertile compared to the lowest tertile (Supporting Information S1: Table 4). To assess potential confounding, we compared demographic and clinical characteristics across exposure tertiles, compared to infants in the lowest tertile of exposure, infants in the highest tertile of exposure were more likely to be diagnosed with asthma, be male, born 2014 or before, receive public insurance, be diagnosed with grade 1 BPD, and not require supplemental respiratory support at time of NICU discharge (Supporting Information S1: Table 5).

## 4 Discussion

In this retrospective cohort of 337 children with BPD, we found that average ambient PM_2.5_ exposure during the first 12 months after NICU discharge was associated with asthma diagnosis by 5 years of age. The observed associations remained robust after adjustment for clinical neonatal factors, socio‐economic factors, and race/ethnicity. Importantly, our results build upon evidence that links ambient PM_2.5_ to wheeze, respiratory deficits, and asthma in infants born preterm [22, 30], and demonstrate that, among children with compromised lung function, air pollution may influence asthma susceptibility later on in childhood. Specifically, our findings add to the small but growing literature suggesting that post‑discharge PM_2.5_ exposure may influence long‐term respiratory outcomes among infants with BPD.

The impacts of air pollution exposure on preterm infant lung health have been previously studied. For instance, a study by Decrue et al. [33] found that children born preterm had lower FEV_1_ than healthy controls and that prenatal exposure to air pollutants, such as PM_2.5_, PM_10_, and NO_2_, can further impair postnatal lung function. They note that preterm infants may have a reduced capacity to handle oxidative stress, making them especially susceptible to respiratory inflammation and long‐term lung impairment from pollution. However, while these findings corroborate our observed associations, the study relied on prenatal air pollution exposure to estimate postnatal effects.

Few studies isolate postnatal ambient air pollutant exposures as the primary exposure of interest, and those that do typically encompass short‐term exposures or short‐term outcomes. Our findings are consistent with a study by Kelchtermans et al. [17] in which short‑term post‐discharge pollution exposures among infants with BPD were associated with emergency department visits with systemic steroid administration. These findings were similar to our prior studies demonstrating associations of the CDC's environmental justice index and PM_2.5_ in the year after NICU discharge with medically attended acute respiratory illness (i.e., an ED visit or inpatient readmission) in the first year after NICU discharge among infants with BPD [8, 34] [9, 14]. However, these studies focused on acute episodes rather than how children fared in terms of long‐term respiratory outcomes. Our research builds upon these findings by examining early‐life ambient PM_2.5_ exposure with a longer‐term outcome, asthma diagnosis by age 5.

Our findings are biologically plausible. Lungs of preterm infants can exhibit limited alveolarization, dysregulated angiogenesis, and heightened oxidative stress [35, 36]. Ultrafine and fine particles penetrate distal airways, generate reactive oxygen species, and amplify Th2‑skewed inflammation, pathways that are already primed in infants with BPD [37, 38]. Models that categorized PM_2.5_ in tertiles revealed an exposure dose–response gradient, with the highest tertile of PM_2.5_ exposure conferring a significantly increased asthma risk among children compared to the lowest tertile of PM_2.5_ exposure. This dose–response relationship is consistent with biological synergies reflected in a 2025 study of asthma in Fresno, California, Cisneros et al., in which PM_2.5_ exposure above 9 μg/m^3^, the current standard set by the Environmental Protection Agency (EPA), over 14 days was associated with increased risk of an acute care visit for asthma [29, 39]. Average annual PM_2.5_ exposure in the cohort was less than the current EPA threshold of 9 μg/m^3^ [29]_._ Therefore, it is important to note that the adverse health effects of fine PM exposure may remain even at relatively low concentrations. Further, individual factors such as birth weight, gestational age, and BPD diagnosis have been shown to be associated with asthma risk in children [24, 25, 28]. Specifically, in a study of 131,783 children hospital with asthma in Denmark, Sweden, and Finland, Liu et al. reported that risk of childhood asthma hospitalization increased with each 1000 g decrease in birth weight (RR 1.17; 95% CI: 1.15, 1.18) and with each decrease in gestational age (RR 1.05; 95% CI: 1.04, 1.06) [28]. We detected an association of a potentially modifiable environmental exposure associated with increased risk of asthma, even when adjusting for individual clinical characteristics. Importantly, the effect sizes highlight that even small reductions in average exposure (e.g., 1 µg/m^3^) may provide meaningful long‐term respiratory benefits for children with BPD. Findings that have important implications for health policy and law as it pertains to air quality and the long‐term health outcomes of medically vulnerable infants and children.

### 4.1 Strengths and Limitations

Our study had several strengths. We utilized a cohort of infants with Grade 1–3 BPD with continued follow‐up in the CHOP Care Network through the age of 5. Ambient PM_2.5_ was obtained from a high‑resolution spatiotemporal machine learning XIS model and aggregated at the census block group level. We employed robust adjustment for clinical, demographic, and socioeconomic factors, in combination with sensitivity analyses, to strengthen the internal validity of our findings. In future studies, effort should be allocated to studies with personal air‑pollution monitors and where household triggers are captured, and whether other morbidities mediate the association presented here.

This study has several limitations that warrant consideration. PM_2.5_ exposure estimates used in these analyses did not capture indoor or personal environments, including exposures to secondhand smoke; therefore, non‑differential misclassification of the exposure is plausible. Furthermore, our observed associations are likely influenced by survivor and referral biases as only infants surviving to discharge and returning for follow‑up within the CHOP Care Network were studied. The impact of these biases can result in potentially under‑representing the sickest or most socio‑marginalized children. ICD‐9/10 codes may lead to outcome misclassification; however, we utilized a two‐step approach including manual chart reviews within the EHR to minimize this limitation. However, a provider diagnosed asthma by age 5 remains a proxy for respiratory symptoms and not an objective measure, such as performance on a pulmonary function test. Therefore, whether these findings represent true biologic asthma versus a childhood manifestation of BPD remains unclear. Infants included in the present study resided in one geographic region with its own PM2.5 exposure trends, and from one healthcare system with its unique practice variations; therefore, these findings may not be generalizable to other parts of the country and warrant further study. The associations presented here were estimated across a heterogeneous cohort spanning multiple counties in one geographic region. To address potential biases arising from differential referral patterns and clinical sites, we performed sensitivity analyses based on discharge address in Philadelphia County and outside of Philadelphia County. Although these models yielded similar effect estimates in both the magnitude and directionality of the associations, the smaller sample size likely accounts for the imprecise estimates.

## 5 Conclusion

We found associations of ambient PM_2.5_ exposure during the first 12 months after NICU discharge with asthma by age 5 among infants with BPD. Our analyses were robust to adjustment for clinical and socioeconomic confounders and exhibited a potential dose‐response relationship. These findings suggest that creating guidance to decrease fine PM exposure during a pivotal phase of lung growth may offer an actionable pathway to mitigate long‑term respiratory morbidity among high‐risk neonates with BPD. Given that environmental exposures may be modifiable with personal exposure mitigation and environmental policy, these results have important implications for developing targeted interventions to improve long‐term health outcomes in children with BPD.

## Author Contributions

Lizbeth F. Gomez, Timothy D. Nelin, and Heather H. Burris conceptualized and designed the study, coordinated, supervised, and collected data, carried out analysis, interpreted the data, and analyzed the results. Lizbeth F. Gomez wrote the initial manuscript draft and reviewed and revised the manuscript throughout the submission process. Timothy D. Nelin and Heather H. Burris reviewed and revised the initial draft and were involved with revisions throughout the submission process. Jonathan J. Szeto, Joshua K. Radack, Sara B. DeMauro, Chén C. Kenyon, Nicolas P. Novick‐Goldstein, Kristan A. Scott, Daria C. Murosko, Kathleen A. Gibbs, Ella Whitman, Jonathan C. Levin, Allan C. Just, and Scott A. Lorch helped conceptualize the study, reviewed, and revised the manuscript throughout the submission process.

## Funding

L.F.G. supported by NHLBI R01HL162354; T.D.N. supported by NIH T32 HL160493.

## Conflicts of Interest

The authors declare no conflicts of interest.

## Supporting information

AsthmaPMBPD_Supplements.docx.

## Data Availability

The data sets generated during/or analyzed during the current study and the code used to analyze and manage the data are available from the corresponding author on reasonable request.
