# Supplementary material for: Associations of Early Life Ambient PM2.5 Exposure With Asthma Risk in a Cohort of Preterm Infants With Bronchopulmonary Dysplasia
Source: Pediatr Pulmonol. 2025 Dec 17;60(12):e71432. doi: 10.1002/ppul.71432 (PMC12710147; doi:10.1002/ppul.71432)
Supplement: Supplementary file 1 — AsthmaPMBPD_Supplements.docx. [file PPUL-60-0-s001.docx]

**SUPPLEMENTAL MATERIALS FOR MANUSCRIPT**

***Tables***

**Supplemental Table 1.** Characteristics of infants with a discharge address in the metropolitan Philadelphia region without documented follow-up through age 5 in the CHOP Care Network.

| **Infant Characteristic** | **Excluded**  **(n = 41)** | **Included**  **(n = 337)** | ***p*-value** |
| --- | --- | --- | --- |
| Gestational Age, weeks, median [IQR] | 28 [25–32] | 27 [25-28] | 0.03 |
| Birth Weight, grams, median [IQR] | 802 [660–1395] | 771 [630-1015] | 0.12 |
| Male sex, n (%) | 21 (51) | 199 (59) | 0.34 |
| Grade 2/3 BPD, n (%) |  |  | 0.06 |
| Grade 2 | 20 (49) | 133 (39) |  |
| Grade 3 | 14 (34) | 84 (25) |  |
| Discharge age, days, mean (SD) | 158 (115) | 156 (89) | 0.90 |
| No Respiratory Support at Time of NICU Discharge, n (%) | 29 (71) | 251 (74) | 0.83 |
| No Inhaled Medications at Time of NICU Discharge, n (%) | 20 (48) | 251 (74) | 0.12 |
| Public Insurance, n (%) | 27 (66) | 208 (62) | 0.61 |
| Community Material Deprivation Index, mean (SD) | 0.33 (0.15) | 0.41 (0.16) | <0.01 |
| PM_2.5_, mean (SD), μg/m^3^ | 8.3 (0.8) | 8.8 (1.1) | <0.01 |

| **ICD-10 Code** | **Description** | **ICD-9 Code** | **Description** |
| --- | --- | --- | --- |
| J45.20 | Mild intermittent asthma, uncomplicated | 493.00 | Extrinsic asthma, unspecified |
| J45.21 | Mild intermittent asthma with (acute) exacerbation | 493.01 | Extrinsic asthma with status asthmaticus |
| J45.22 | Mild intermittent asthma with status asthmaticus | 493.02 | Extrinsic asthma with (acute) exacerbation |
| J45.30 | Mild persistent asthma, uncomplicated | 493.10 | Intrinsic asthma, unspecified |
| J45.31 | Mild persistent asthma with (acute) exacerbation | 493.11 | Intrinsic asthma with status asthmaticus |
| J45.32 | Mild persistent asthma with status asthmaticus | 493.12 | Intrinsic asthma with (acute) exacerbation |
| J45.40 | Moderate persistent asthma, uncomplicated | 493.20 | Chronic obstructive asthma, unspecified |
| J45.41 | Moderate persistent asthma with (acute) exacerbation | 493.21 | Chronic obstructive asthma with status asthmaticus |
| J45.42 | Moderate persistent asthma with status asthmaticus | 493.22 | Chronic obstructive asthma with (acute) exacerbation |
| J45.50 | Severe persistent asthma, uncomplicated | 493.81 | Exercise-induced bronchospasm |
| J45.51 | Severe persistent asthma with (acute) exacerbation | 493.82 | Cough-variant asthma |
| J45.52 | Severe persistent asthma with status asthmaticus | 493.90 | Asthma, unspecified |
| J45.909 | Unspecified asthma, uncomplicated | 493.91 | Asthma, unspecified with status asthmaticus |
| J45.901 | Unspecified asthma with (acute) exacerbation | 493.92 | Asthma, unspecified with (acute) exacerbation |
| J45.902 | Unspecified asthma with status asthmaticus |  |  |

**Supplemental Table 2.** Asthma ICD-9/10 codes used in data query.

**Supplemental Table 3**: Distribution of infants with a discharge address in Philadelphia County and outside of Philadelphia County by PM_2.5_ tertile.

| County | Overall (n = 337)  n (%) | PM_2.5_ Tertile 1  (n= 113)  n (%) | PM_2.5_ Tertile 2  (n= 112)  n (%) | PM_2.5_ Tertile 3  (n = 112)  n (%) |
| --- | --- | --- | --- | --- |
| Philadelphia | 159 (47) | 44 (39) | 56 (50) | 59 (52) |
| Non-Philadelphia ^a^ | 178 (53) | 69 (61) | 56 (50) | 53 (47) |

^a^ Non-Philadelphia Counties: Pennsylvania – Berks, Bucks, Chester, Delaware, Montgomery; New Jersey – Atlantic, Burlington, Camden, Cape May, Cumberland, Gloucester, Mercer; Delaware – Cumberland, Kent, New Castle

**Supplemental Table 4.** Poisson regression models quantifying the association of mean annual PM_2.5_ exposure during the first 12 months post–NICU discharge with asthma diagnosis by age five among infants with BPD in Philadelphia County and outside of Philadelphia County.

| **PM_2.5_ Exposure** | **Adjusted Model^a^, Philadelphia County**  **(n = 146)** | | **Adjusted Model^a^,**  **Non-Philadelphia County**  **(n = 178)** |
| --- | --- | --- | --- |
|  | aRR (95% CI) | | aRR (95% CI) |
| Continuous  (per 1 µg/m^3^) | 1.14 (0.94, 1.38) | 1.19 (0.94, 1.53) | |
| Tertile 1 ^b^ | Reference | Reference | |
|  |  |  | |
| Tertile 2 ^c^ | 1.47 (0.99, 2.17) | 1.00 (0.59, 1.69) | |
|  |  |  | |
| Tertile 3 ^d^ | 1.56 (0.88, 2.77) | 1.63 (0.92, 2.87) | |

*Abbreviations:* aRR = adjusted risk ratio; PM_2.5_ = fine particulate matter with aerodynamic diameter ≤ 2.5 μm.

Estimates obtained using Poisson regression with robust standard errors.

ᵃ Adjusted for gestational age, birth weight, birth period, sex, BPD grade, discharge age, discharge support, discharge medication, insurance type, census-tract neighborhood community material deprivation, race/ethnicity.

^b^ Tertile 1 includes 44 infants in Philadelphia County (mean PM_2.5_ 7.6 µg/m^3^ ) and 69 infants outside of Philadelphia County (mean PM_2.5_ 7.6 µg/m^3^).

^c^ Tertile 2 includes 56 infants in Philadelphia County (mean PM_2.5_ 8.7 µg/m^3^) and 56 infants outside of Philadelphia County (mean PM_2.5_ 8.7 µg/m^3^).

^d^ Tertile 3 includes 59 infants in Philadelphia County (mean PM_2.5_ 10.1 µg/m^3^) and 53 infants outside of Philadelphia County (mean PM_2.5_ 9.8 µg/m^3^).

**Supplemental Table 5.** Demographic baseline characteristics of included infants stratified by tertiles of exposure to PM_2.5_

| Characteristic | Tertile 1  PM_2.5_ ≤7.6 μg/m^3^ | Tertile 2  PM_2.5_ 7.6 - 8.7μg/m^3^ | Tertile 3  PM_2.5_ ≥8.7 μg/m^3^ |  |
| --- | --- | --- | --- | --- |
| Demographic features | n=113 | n = 112 | n = 112 |  |
| Sex (Male) — n (%) | 63 (55.8) | 56 (50.0) | 80 (71.4) |  |
| Birth period > 2014 — n (%) | 105 (92.9) | 86 (76.8) | 16 (14.3) |  |
| Insurance — n (%) |  |  |  |  |
| Commercial | 52 (46.0) | 39 (34.8) | 38 (33.9) |  |
| Public | 61 (54.0) | 73 (65.2) | 74 (66.1) |  |
| Race/Ethnicity — n (%) |  |  |  |  |
| Non-Hispanic Black | 53 (46.9) | 60 (53.6) | 61 (54.5) |  |
| Non-Hispanic White | 38 (33.6) | 21 (18.8) | 29 (25.9) |  |
| Other | 22 (19.5) | 31 (27.7) | 22 (19.6) |  |
| Clinical features |  |  |  |  |
| Asthma at age 5 — n (%) | 45 (39.8) | 58 (51.8) | 66 (58.9) |  |
| Gestational Age — Weeks, Mean (SD) | 27 (2.4) | 27 (2.0) | 26 (2.5) |  |
| Extremely preterm — n (%)¶ | 72 (63.7) | 81 (72.3) | 74 (66.1) |  |
| Birth weight — Grams, Mean (SD) | 863.5 (310.6) | 816 (273.9) | 876 (332.8) |  |
| ELBW^+^ — n (%) | 81 (71.7) | 88(78.6) | 80 (71.4) |  |
| BPD Grade — n (%)^#^ |  |  |  |  |
| Grade 1 | 33 (29.2) | 38 (33.9) | 49 (43.8) |  |
| Grade 2 | 48 (42.5) | 43 (38.4) | 42 (37.5) |  |
| Grade 3 | 32 (28.3) | 31 (27.7) | 21 (18.8) |  |
| Readmission in year after NICU discharge — n (%) | 54 (47.8) | 52 (46.4) | 58 (51.8) |  |
| Discharge age — Days, Mean (SD) | 178 (94.5) | 150 (93.0) | 138 (74.6) |  |
| Medications at NICU Discharge — n (%) |  |  |  |  |
| Albuterol | 26 (23.0) | 16 (14.3) | 16 (14.3) |  |
| Albuterol/Inhaled Corticosteroid | 9 (8.0) | 10 (8.9) | 8 (7.1) |  |
| Discharge respiratory support — n (%) |  |  |  |  |
| No support | 84 (74.3) | 80 (71.4) | 87 (77.7) |  |
| Supplemental Oxygen | 7 (6.2) | 15 (13.4) | 18 (16.1) |  |
| Tracheostomy | | 22 (19.5) | 17 (15.2) | 7 (6.2) |
| Abbreviations: ELBW, Extremely Low Birth Weight <1000 grams; BPD, bronchopulmonary dysplasia; NICU, neonatal intensive care unit  ¶ Extremely Preterm correspond to infants born before 28 weeks of gestation.  ^#^BPD grade criteria defined by the 2019 Neonatal Research Network. Grade 1 = nasal cannula at a flow of ≤2 L/min; Grade 2 = nasal cannula at a flow of >2 L/min or noninvasive positive airway pressure; and Grade 3 = to infants requiring invasive mechanical ventilation. | | | |  |

***Figures***


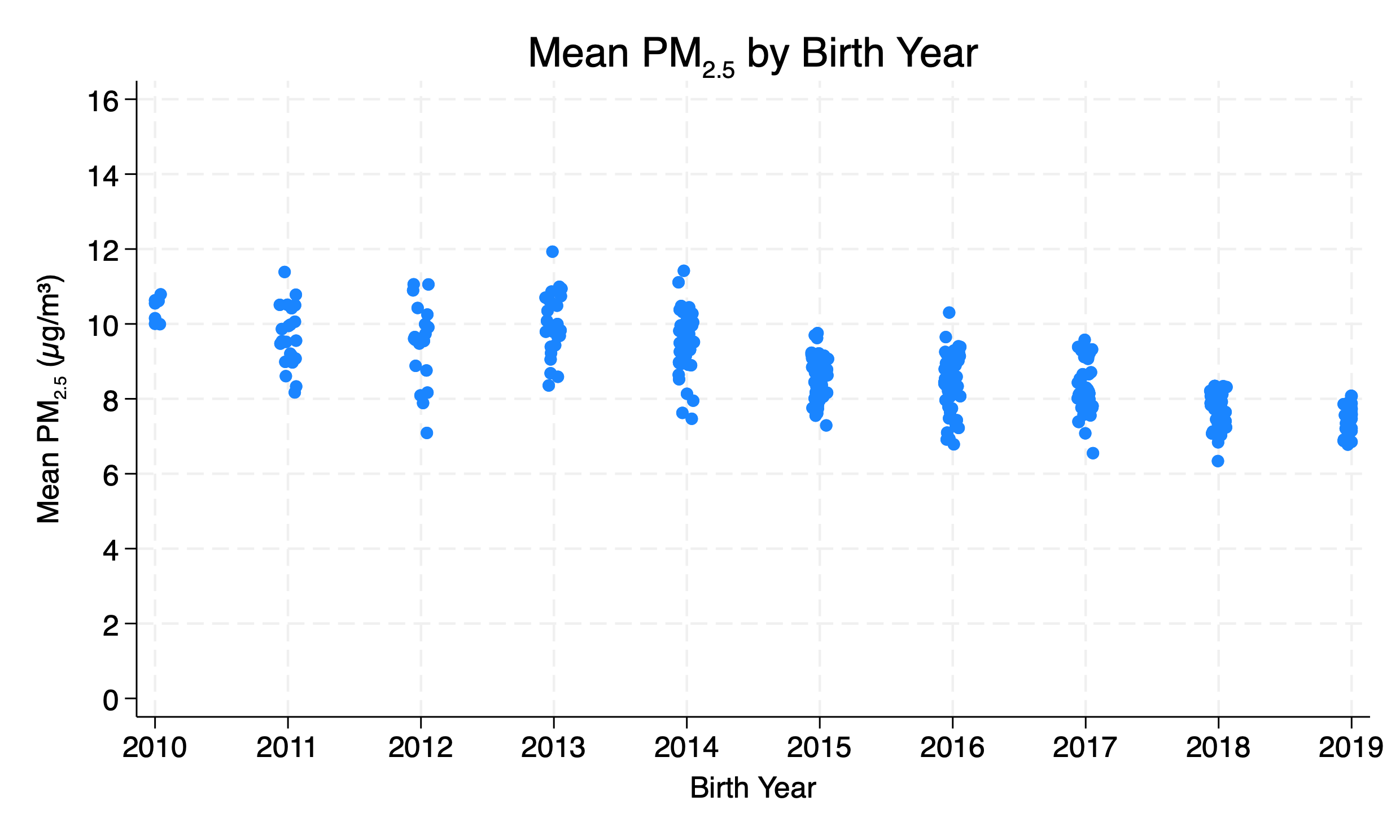


**Supplemental Figure 1**: Distribution of annual PM_2.5_ from across birth year (2010-2020)
